# Protein quality malnutrition

**DOI:** 10.3389/fnut.2024.1428810

**Published:** 2024-10-23

**Authors:** Mark J. Manary, Donna R. Wegner, Kenneth Maleta

**Affiliations:** ^1^Department of Pediatrics, Washington University, St. Louis, MO, United States; ^2^Children’s Nutrition Research Center, USDA, Houston, TX, United States; ^3^Department of Nutrition and Dietetics, School of Global and Public Health, Blantyre, Malawi

**Keywords:** protein quality, protein metabolism, malnutrition, PDCAAS, DIAAS

## Abstract

Protein quality refers to the evaluation of a food or a diet based on its amino acid composition, protein digestibility, and protein bioavailability. When these parameters are specified, either through direct measurement or estimation, the amino acids provided by the diet are compared to those required by a healthy individual, and based on this comparison, an adequacy ratio or score is assigned. Two widely used protein quality scoring systems are the protein digestibility-corrected amino acid score (PDCAAS) and the digestible indispensable amino acid score (DIAAS), neither of which account for the dietary source of the protein. In malnourished children, metabolic adaptations reduce the endogenous availability of amino acids and increase the demand for protein synthesis. These increased amino acid requirements are primarily driven by the presence of acute infection and the need for tissue accretion. This review examines two large clinical feeding trials involving moderately malnourished children, where dietary protein quality was carefully measured. The finding s suggest that protein quality scores alone do not reliably predict weight gain or recovery in these children and that consuming milk protein provides distinct advantages over vegetable-based proteins.

## Introduction

Protein quality refers to the evaluation of a food or a diet based on its amino acid composition, protein digestibility, and protein bioavailability (1). Amino acid composition refers to the amounts of each amino acid present in the food. There are 20 different amino acids, 9 of which are classified as essential because they cannot be synthesized by the human body, while the remaining 11 are non-essential and can be synthesized in limited quantities.

Since the interconversion of one amino acid to another is quite limited, essential amino acids function as independent, essential nutrients (2). Digestibility refers to the ability of the human digestive tract to denature proteins and enzymatically or chemically break them into smaller peptides or individual amino acids. Animal-based proteins are typically more digestible, while plant-based proteins may form aggregates that resist digestion (3).

Bioavailability refers to the extent to which digested amino acids and small peptides are absorbed in a form that supports protein synthesis. Food processing and cooking generally enhance both the digestion and bioavailability of dietary protein. Inadequate protein quality, or protein quality malnutrition, occurs when a diet chronically fails to deliver enough amino acids to the systemic circulation to sustain the wide range of physiological functions needed for optimal health.

Two widely used protein quality scoring systems are the protein digestibility-corrected amino acid score (PDCAAS) and the more recently developed digestible indispensable amino acid score (DIAAS) (4, 5). The DIAAS provides a more accurate assessment of the three components of protein quality: amino acid composition, digestibility, and bioavailability. Both scoring systems identify the limiting amino acid in a given food or diet, which restricts the potential for protein synthesis.

This approach assumes that the amino acid requirements are similar to those of a healthy individual. Studies involving animals raised for meat have validated this assumption by demonstrating that the addition of the limiting amino acid results in significant gains in lean body mass in growing animals (6). Due to this robust evidence, most animals raised for meat in the current times are fed diets that are fortified with up to five additional amino acids to improve the protein quality score. However, a key limitation of both scoring systems is the inaccurate assumption that all consumers share uniform physiological states.

### Anatomy, physiology, and biochemistry of protein metabolism

The small intestine is divided into two anatomic sections: the duodenum/jejunum, which contains fewer microbes, and the ileum, which hosts a large and highly active microbial population. Amino acid digestion in the duodenum/jejunum is primarily enzymatic, with absorption occurring via distinct transmembrane transport complexes. In the ileum, mammalian enzymatic digestion continues, but microbes may both catabolize and synthesize certain amino acids. Microbial amino acids may also be absorbed. While the ileum is not the primary site of amino acid absorption, it can serve as an important source of essential amino acids that are not directly derived from the diet.

Protein synthesis and degradation are continuous and dynamic processes in the human body. Unlike other nutrients, amino acids are not stored in reservoirs, but the constant degradation of proteins provides a steady source of amino acids for new protein synthesis. During periods of increased protein demand, such as in response to acute infection, muscle proteins undergo proteolysis to supply the necessary amino acids for enhanced protein synthesis.

Dietary amino acid requirements include both the amino acids necessary for maintaining basic cellular functions and those required to meet special physiological demands. In low-resource settings, children often experience three particular physiological conditions that significantly affect their dietary protein needs: concurrent acute infection, chronic illness, and the demand of growth and development (7). During an acute infection, amino acid requirements typically increase by approximately 50% to support the body’s needs (8).

During growth, the amino acid requirements increase by 50–100%, as more amino acids are required to generate new tissue compared to the amount needed for maintaining existing tissue (9). The most common chronic illnesses associated with increased dietary amino acid requirements are those characterized by excessive inflammation, such as HIV or tuberculosis. These infections are estimated to raise amino acid requirements by 10–20% (10). Notably, new tissue accretion cannot occur simultaneously with the body’s response to an acute infection, as these processes compete for amino acids. Some nutritionists have speculated that chronic inflammation of the small bowel, commonly known as environmental enteric dysfunction (EED), might impair amino acid absorption. However, recent isotopic studies indicate that amino acid absorption is not compromised in cases of EED (11).

### Malnutrition and protein metabolism

**Figure 1:**
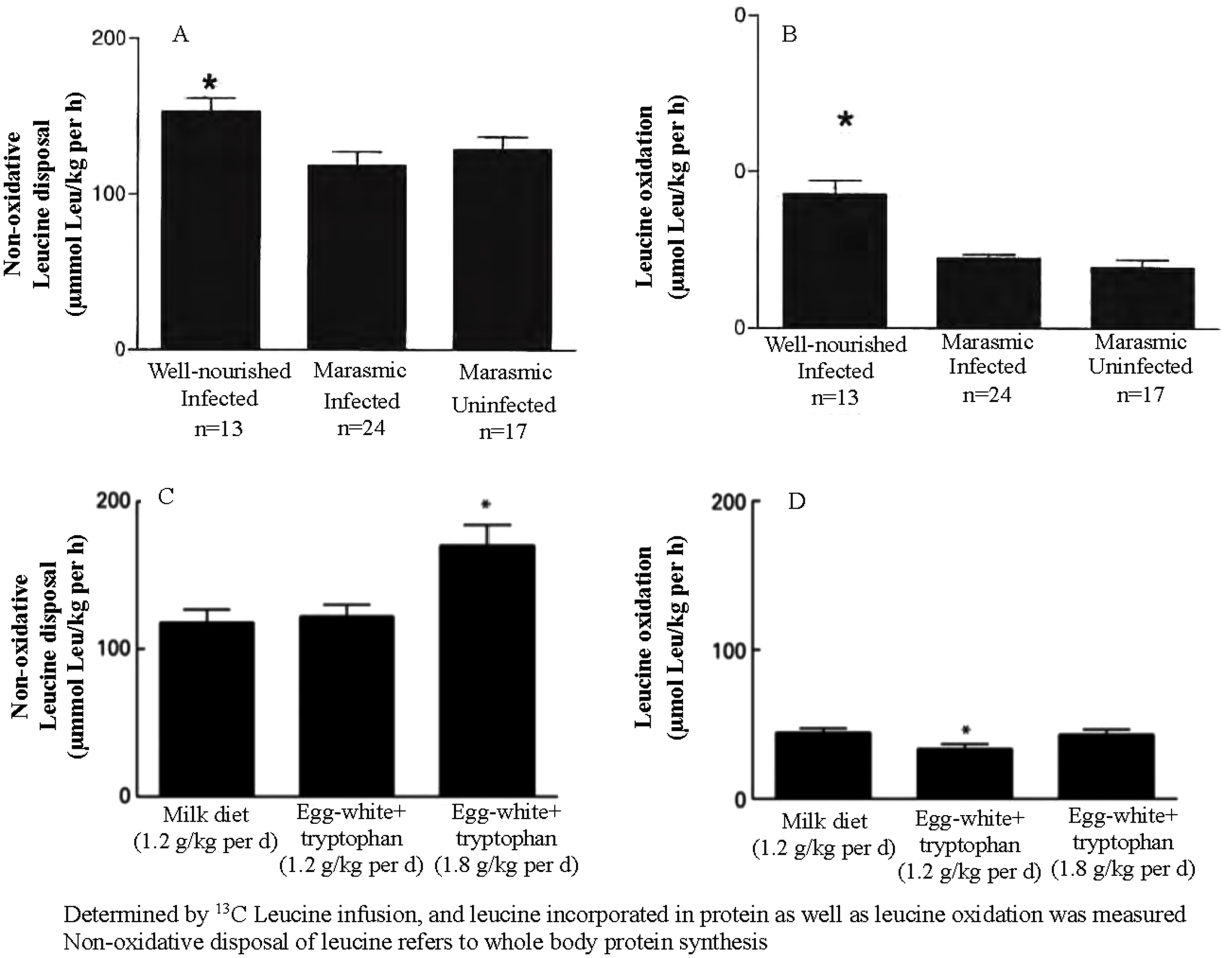
**(A, B)** Represent all malnourished children. **(C, D)** Compare protein kinetics in children receiving one or two differing amounts and types of protein.

The metabolic response to malnutrition involves a reduction in protein kinetics, which helps conserve amino acids and other nutrients that are in limited supply. However, this adaptation also decreases the body’s capacity to respond to acute infection, thereby increasing the risk of poorer outcomes. This was evident in malnourished Malawian children approximately 20 years ago (12–17). As shown in Figure 1A, well-nourished children with infection exhibit higher rates of whole-body protein synthesis than wasted children with infection, even when both groups are fed an isonitrogenous and isoenergetic diet. Figure 1B shows that amino acid oxidation is also elevated in well-nourished children. In malnourished children, the metabolic protein kinetic response is blunted, as evidenced by similar rates of whole-body protein synthesis in wasted children with and without infection.

The milk and egg white + tryptophan diets were both isonitrogenous and isoenergetic, although the egg white + tryptophan diet had a higher protein quality score. As shown in Figure 1C, whole-body protein synthesis is comparable between the two diets and increases with higher protein intake. Figure 1D demonstrates that the diet with the higher protein quality score resulted in a lower rate of amino acid oxidation, as the dietary amino acids more closely matched the body’s response to acute infection. These metabolic studies support the practice of providing malnourished children with food aid products with high protein quality scores and generous amounts of animal-based protein.

### Clinical trial evidence regarding protein quality

**Figure 2:**
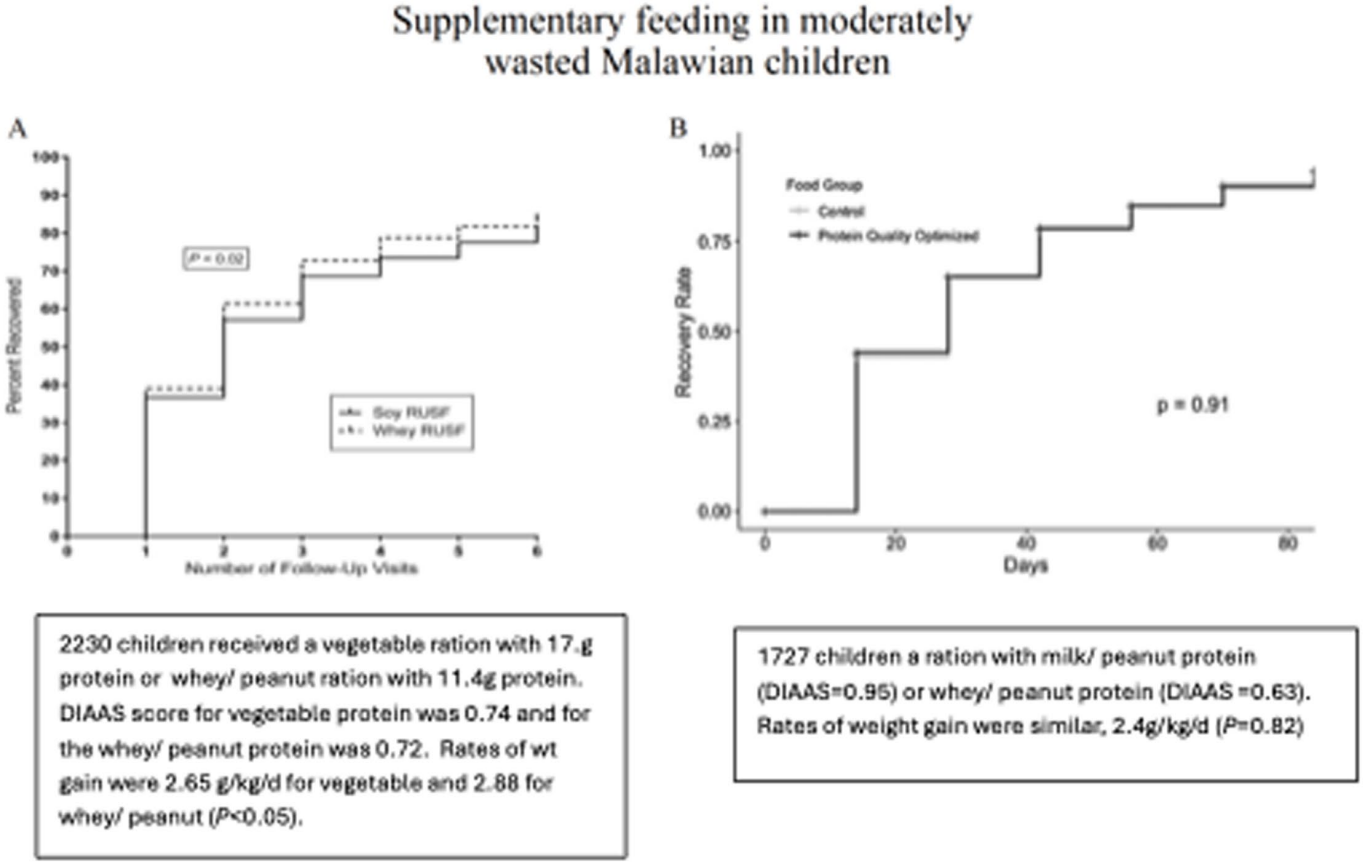
In panel **A**, 2220 children received a daily ration that contained 17.1 g vegetable protein or 11.4g whey/ peanut protein. DIASS score for vegetable protein 0.74 and 0.72 for the whey/ peanut. Rates of wt gain 2.88 g/kg/d whey vs 2.65 (*P*< 0.05). In panel **B**, 1727 children received a ration peanut/ milk protein (DIAAS=0.95) or peanut/ whey protein (DIAAS=0.63). The rates of weight gain were 2.44 g/kg/d and 2.40 g/kg/d.

Two trials involving food aid products in malnourished children, where protein quality was controlled, suggest that the food source of dietary protein—beyond just its quality—may also play a role in determining clinical outcomes. Both trials were conducted among Malawian children with moderate wasting. These two trials used peanut-based ready-to-use supplementary foods (RUSFs). The results from the first trial are shown in Figure 2A, where similar RUSFs were made with either soy protein or whey protein (18). The formulations were adjusted to achieve similar DIAAS of approximately 0.73. In order to achieve this score, the soy formulation included 17.1 g of the total protein, while the whey formulation included 11.4 g of the total protein. All other nutrients were provided in similar quantities. Rates of recovery and weight gain were higher in the children who received the whey formulation.

Figure 2B shows the results comparing two RUSFs containing different types of dairy protein: whey or milk (19). The DIAAS of the whey RUSF was 0.63 and that of the milk RUSF was 0.95. This study was unique in that the DIAAS was measured using a growing pig model, which requires feeding the test diet for 1 week. Other determinations of the DIAAS were made by calculations from previous experiments. Rates of recovery rate and weight gain were identical among the 2,200 study children. These two studies suggest that in malnourished children, the source of the protein is a determinant of clinical outcomes, irrespective of the protein quality score. Dairy protein, particularly milk protein, appears to confer a benefit.

It has been established that the DIAAS correlates well with *in vivo* measures of protein utilization in adult humans. However, the measurement of growth may not be sensitive enough to detect such differences. Furthermore, the amino acid score for a healthy child may not correspond well with that of a malnourished child, and dietary factors other than absorbed amino acids may affect recovery.

## Discussion

Studies on animals provide strong evidence for the importance of the protein quality score in a homogenous population—one that is healthy and free from acute infections or varying degrees of wasting. These studies focus on a specific outcome: the accretion of lean body mass. Therefore, the assumption regarding the amino acid requirements of the reference population, which serves as the denominator of the protein quality score, is more likely to be valid.

In contrast, among moderately wasted children, there is more dynamic variation in the nature and duration of intercurrent infectious illnesses, as well as in the degree of catch-up growth required for recovery. These variations affect each child’s amino acid requirement, making the determination of the DIAAS less precise. Empirical protein kinetic data are needed to more accurately determine a protein quality score for this population.

One of the goals of developing protein quality scores is to compare highly diverse foods and diets and predict their effects on human nutrition. Data from moderately wasted children suggest that milk protein is superior to whey protein, which is superior to soy protein. Milk protein contains much more casein than whey, and casein is known to be a source of bioactive peptides (20–22).

These peptides are created by the hydrolysis of casein during ingestion, rather than existing as distinct minor components of milk. There are over 700 casein-derived bioactive peptides. Their activities include anti-inflammatory, antioxidant, and antimicrobial effects. These peptides exert their effects through specific receptors in the gut. The presence of both casein and whey proteins in all mammalian milk suggests that they have been conserved through evolution to provide benefits to immature, growing mammals. The potential benefits of these bioactive milk peptides for malnourished children should be considered when evaluating the source of dietary protein, in addition to the protein quality score of the diet.

Additionally, the DIAAS allows for additivity and complementarity of dietary proteins in a meal. Lower-quality proteins, or proteins with limiting IAAs, can be complemented by higher-quality proteins, or proteins in which the limiting amino acid is present in excess, resulting in an overall higher protein quality meal. This property has driven an agenda advocating plant-based proteins as alternatives to animal-based proteins, largely due to concerns regarding the environmental impact of animal protein production. However, as demonstrated above, such considerations should be approached with caution in populations with high levels of malnutrition, where both protein quality and protein source may be critical to preventing protein quality malnutrition.

## Data Availability

The original contributions presented in the study are included in the article/supplementary material, further inquiries can be directed to the corresponding author.
